# Donor Microbiota Composition and Housing Affect Recapitulation of Obese Phenotypes in a Human Microbiota-Associated Murine Model

**DOI:** 10.3389/fcimb.2021.614218

**Published:** 2021-02-22

**Authors:** Thomas Kaiser, Harika Nalluri, Zhigang Zhu, Christopher Staley

**Affiliations:** ^1^ Division of Basic & Translational Research, Department of Surgery, University of Minnesota, Minneapolis, MN, United States; ^2^ Biotechnology Institute, University of Minnesota, Saint Paul, MN, United States

**Keywords:** fecal microbiota transplantation, human microbiota-associated, mouse model, obesity, specific pathogen-free, conventional housing

## Abstract

Human microbiota-associated (HMA) mouse models offer a valuable approach to study the role of intestinal microbiota in the development of obesity. In this study, we used an HMA model to evaluate whether engraftment of human obese or lean microbiota, from each of three donors, could recapitulate host phenotypes under conventional and specific-pathogen-free housing. Microbiota engraftment was correlated with donor relative abundances of the class Bacteroidia (Spearman’s *ρ* = 0.73, *P* ≤ 0.001), and one obese donor resulted in significant weight gain (*P* ≤ 0.003) and compromised insulin sensitivity under conventional housing. SPF housing partially blunted phenotypic response. Results of this study indicate that our HMA model partially recapitulates obese phenotypes under conventional housing and highlights a need to consider donor-specific effects as well as housing conditions when studying the role of the microbiota in obesity.

## Introduction

Obesity and obesity-related metabolic disorders are major public health concerns with rising prevalence (Chooi et al., 2019). The intestinal microbiota has recently been implicated as playing a key role in physiologic homeostasis and energy metabolism. Differences in gut microbiota have been identified between obese and lean humans (Turnbaugh et al., 2009a; Ley, 2010). Furthermore, transfer of the intestinal microbiota from twins discordant for obesity led to a recapitulation of the human phenotype in germ-free (GF) mice (Ridaura et al., 2013), demonstrating a likely causal role of the microbiota in obesity and metabolic dysregulation. Correction of the microbiota by fecal microbiota transplant of lean microbiota was also shown to improve insulin sensitivity in patients with metabolic syndrome (Vrieze et al., 2012; Kootte et al., 2017). While these findings are promising, elucidating additional mechanistic insights will be crucial to develop novel therapies to treat obesity.

Human microbiota-associated (HMA) animal models have become an invaluable tool to study human microbiota in a controlled and modifiable experimental setting. Traditionally, this involves the transfer of human microbiota into GF mice bred in isolators to prevent exposure to microorganisms (Turnbaugh et al., 2009b). The GF mouse, however, is increasingly recognized as a poor model for clinical translation due primarily to the lack of a developed immune system (Masopust et al., 2017), as well as less immune activation following transplantation of human microbiota relative to murine (Lundberg et al., 2020). Specific-pathogen-free (SPF) mice have similarly received criticism as the hygienic barriers to SPF housing have greatly altered the microbiota in these colonies, with similar immunological and clinically translational weaknesses (Dobson et al., 2019). These discrepancies between housing have resulted in differences in physiologic outcomes. In one study, rats kept under conventional housing showed better responses to surgical trauma than those in SPF housing (Letson et al., 2019). As the microbiota is increasingly studied as an underlying component of chronic diseases like obesity, characterizing differences in composition and host phenotypic response due to housing is critical.

To account for weaknesses in physiology as well as accessibility in the GF model, alternative HMA models in non-GF animals have been pursued that involve microbial depletion of indigenous microbiota by antibiotics prior to repopulation (Wos-Oxley et al., 2012; Hintze et al., 2014; Staley et al., 2017; Daharsh and Ramer-tait, 2019; Rodriguez et al., 2019). However, the majority of these have been done under SPF conditions. Characterizing the impact of housing on host phenotypic response will help optimize the translatability of HMA mouse models. Evidence has suggested that conventional housing, free from barrier or containment restrictions, may more accurately represent a natural environment. Murine obese and lean microbiota transfer under conventional conditions has been possible in the same manner seen in germ-free isolators (Ellekilde et al., 2014). Stable transfer of human microbiota to conventionally housed mice has also been shown (Wrzosek et al., 2018).

In this study, we hypothesized that conventionally housed HMA mice may offer a more translational tool to study microbiota and obesity. Using our previously published HMA protocol (Staley et al., 2017), we tested the potential for engraftment of microbiota from lean or obese human donors alone to drive phenotypic shifts in weight and insulin resistance. Testing was done under conventional and SPF housing to determine how this environmental factor influenced microbiota engraftment and host phenotype. Results from this study highlight how differences in donor microbiota composition impact microbiota engraftment as well as discrepancies in phenotypic shift associated with housing.

## Methods

### Mice and Housing

C57BL/6J mice were purchased at 36–42 days of age from Charles River Laboratories (Wilmington, MA, USA) and were housed at 23°C under 12 h/12 h light-dark cycle. Mice were delivered under SPF conditions and were immediately housed under either SPF conditions (kept in microisolator-filtered cages opened only under a laminar flow hood) or conventional (covered cages without filters) conditions for the remainder of the experiment. Mice were kept on non-autoclaved bedding, fed standard chow (Teklad global 18% protein rodent diet; 18.6% protein, 6.2% fat, 44.2% carbohydrates; Envigo, Indianapolis, IN, USA) *ad libitum*, and provided non-autoclaved water in individual drinking bottles for the duration of the experiment. Bedding and food were not irradiated and were obtained from the same sources for conventional and SPF housing. Mice were housed only with animals of the same sex (maximum five females or four males per cage, per institutional policy). Mice were separated as necessary due to injury or fighting, and any mice that required antibiotic treatment for injuries were euthanized rather than receiving treatment. Individual experimental or control groups were housed separately. All experiments were approved by the University of Minnesota Institutional Animal Care and Use Committee (IACUC), protocol 1801-35494A.

### Antibiotics

Two antibiotic cocktails were prepared in normal drinking water with each antibiotic at a concentration of 1 mg ml^-1^, as previously described (Staley et al., 2017). The systemically absorbed cocktail consisted of ampicillin (WG Critical Care, LLC, Paramus, NJ, USA), cefoperazone sodium salt (Sigma-Aldrich Co., St. Louis, MO, USA), and clindamycin hydrochloride (Fagron, Inc., St. Paul, MN, USA). The non-systemically-absorbed cocktail consisted of ertapenem sodium salt (Merck and Co., Inc., Whitehouse Station, NJ, USA), neomycin sulfate (Fagron, Inc.), and vancomycin hydrochloride (Mylan Institutional LLC, Rockford, IL, USA). Antibiotic solutions were prepared the day prior to administration to mice and stored at 4°C.

### Donor Fecal Preparation

Fecal samples were collected from six Caucasian donors, three lean (Ln) and three obese (Ob; **Supplementary Table S1**). Lean donors were participants of the University of Minnesota Microbiota Therapeutics Donor Program (Hamilton et al., 2012). Exclusion criteria for lean donors included metabolic and autoimmune disorders, history of gastrointestinal diseases or surgery, allergies, neurologic or psychiatric disorders, or use of antibiotics in the prior six months. Obese donors were patients with body mass index (BMI) ≥ 30 kg m^-2^ under clinical supervision at the University of Minnesota Health Comprehensive Weight Management Center. They were enrolled with similar exclusion criteria to lean donors, except for metabolic disorders. All donors provided written consent and were recruited under approval by the University of Minnesota Institutional Review Board (IRB).

Fecal material was prepared as previously described (Hamilton et al., 2012). Briefly, stool was diluted in sterile phosphate-buffered saline, homogenized under N_2_ gas (40 psi), and passed through autoclaved, stainless steel laboratory sieves with a final pore size of 0.25 mm (WS Tyler, Mentor, OH, USA). The material was centrifuged (4°C at 4,500 × *g* for 15 min), supernatant was discarded, and remaining preparation amended with 10% glycerol, using an autoclaved stock of 50% glycerol (glycerol:water), for storage at −80°C. Bacterial concentrations were determined by SYTO™ BC Green fluorescent nucleic acid staining (Invitrogen, Carlsbad, CA, USA), counted in a Petroff Hauser counting chamber, and normalized to a standard dose of 10^10^ cells 100 µl^-1^ (Staley et al., 2017).

### Antibiotic Conditioning and Gavage

The mice received human microbiota *via* our established fecal microbiota transplantation (FMT) protocol, which involves antibiotic conditioning followed by single oral gavage of cryopreserved human fecal microbiota (Staley et al., 2017). Each experiment included mice (*n* = 4 males and 5 females per cohort) that received either obese or lean human donor microbiota, compared to control mice that either received antibiotics alone or received neither antibiotics nor human microbiota (**Supplementary Figure 1**). Mice from each donor pair were tested from separate orders; controls were tested with the first donor pair. Donor pairs sharing the same number were tested using mice from the same order. Mice were cohoused in single male and female cages in each treatment group, but were separated as necessary due to fighting, especially among males, as recommended by the veterinarian.

Mice receiving antibiotic conditioning were given 7-day administrations of the systemically absorbed cocktail, followed by non-systemically-absorbed cocktail, and repeated systemically absorbed cocktail, with 2-day periods of normal drinking water between (Staley et al., 2017). Mice consumed 15–20 ml antibiotic cocktail per day per cage, with reduced consumption of the systemic cocktail. We previously showed that two days off antibiotics does not allow a significant return of indigenous microbiota (Staley et al., 2017). Two days following the final antibiotic cocktail, mice in experimental cohorts received gavage with 100 μl (10^10^ cells) of prepared donor material or normal drinking water, among control groups. The gavage procedure was carried out wearing gloves under conditions appropriate to housing (*i.e.*, in a biosafety cabinet for SPF or under ambient room conditions for conventional) using autoclaved gavage needles. Mice were transferred to fresh bedding following gavage so as not to reintroduce antibiotics or mouse microbiota associated with residual fecal pellets.

### Sample Collection and Monitoring Phenotypic Changes

Fecal pellets were collected prior to antibiotic conditioning (T_pre_), on day of gavage (T_0_), and subsequent four weeks, and were stored at −20°C until DNA extraction. Total body weight was measured weekly from T_0_ to 10-weeks post-gavage (T_10_) and reported as change from baseline to account for cage-specific effects.

When paired obese and lean groups showed significant differences in weight gain, insulin tolerance testing was done at T_10_. After 4 h of fasting, mice were given weight-based intraperitoneal injection of insulin (0.5 U kg^-1^ for females, 1 U kg^-1^ for males). Blood glucose levels were measured in 15-min intervals for 1-h period using Accu-Check^®^ meters and test strips (Roche, Indianapolis, IN). Venous blood was obtained from the tip of the tail in unrestrained mice.

### DNA Extraction and Sequencing

DNA was extracted in triplicate from donor fecal samples and from individual mouse fecal pellets using the DNeasy PowerSoil kit (QIAGEN, Hilden, Germany). The V4 hypervariable region of the 16S rRNA gene was amplified from individual DNA extracts using the 515F/806R primer set (Caporaso et al., 2012) by the University of Minnesota Genomics Center (UMGC, Minneapolis, MN, USA), as described previously (Gohl et al., 2016). Initial cycling conditions were 95°C for 5 min followed by 20 cycles: 98°C for 20 s, 55°C for 15 s, and 72°C for 1 min. Primary amplicons were diluted 1:100 in sterile nucleotide-free water and a second PCR reaction using indexed primers with the same cycling conditions (10 cycles) and a final extension at 72°C for 1 min. Final amplicons were quantified using a PicoGreen dsDNA kit (Life Technologies, Carlsbad, CA, USA) and normalized. Amplicons were size-selected (427 bp ± 20%) on a Caliper XT DNA 750 chip (Caliper Life Science, Hopkinton, MA, USA) and cleaned using AMPureXP beads (Beckman Coulter, Brea, CA, USA). Dual index, paired-end sequencing was done on the Illumina MiSeq Platform (Illumina, Inc., San Diego, CA, USA) by UMGC at a read length of 300 nucleotides (Gohl et al., 2016). This protocol has been previously validated using mock communities (Gohl et al., 2016). Negative (sterile water) controls were included with each sequencing run and did not produce amplicons. Raw data are deposited in the Sequence Read Archive under BioProject accession number SRP166444.

### 16S rRNA Amplicon Processing and Engraftment Analysis

Amplicon sequence data was processed and analyzed using mothur software ver. 1.41.1 (Schloss et al., 2009) and our previously published pipeline for quality screening and taxonomic annotation (Staley et al., 2018). Briefly, reads were paired-end joined, quality trimmed, and aligned against the SILVA database ver. 132 (Pruesse et al., 2007). Operational taxonomic units (OTUs) were binned at 97% similarity using the furthest-neighbor algorithm and taxonomic assignment was done against the version 16 release from the Ribosomal Database Project (Cole et al., 2009). Different databases were used to account for handling of rRNA secondary structure and data masking, as described previously (Schloss and Westcott, 2011). A mean (± standard deviation) of 21,978 ± 14,062 reads per sample were obtained and samples were rarefied to 6,000 reads for statistical comparisons, resulting in a mean estimated Good’s coverage of 98.5 ± 1.3%. A mean of 209 ± 149 (range: 13 – 702) OTUs were observed among all samples following rarefaction.

We used SourceTracker ver. 0.9.8 (default parameters) to assess engraftment of donor microbiota, as the percent of mouse community (sink) that could be attributed to the donor community (source) (Knights et al., 2011). This software uses a Bayesian inference approach to determine what percent of the community in sink samples is derived from source samples. We have previously shown this software provides a conservative estimate of donor engraftment among human FMT recipients (Staley et al., 2019), reporting lower similarity than would be determined by empirically shared OTUs. We designated the human donor and the mouse baseline (prior to antibiotics) microbial communities as sources.

Differences in microbial communities were calculated using the Bray-Curtis dissimilarity index (Bray and Curtis, 1957) and visualized *via* ordination by principal coordinate analysis (PCoA) (Anderson and Willis, 2003). Differences in community composition were evaluated statistically by analysis of similarity (ANOSIM), with Bonferroni correction for multiple comparisons (Clarke, 1993).

### Statistical Analysis

Results are represented as mean ± standard deviation. Data were evaluated between lean and obese arms (and controls) that were run concurrently, to account for differences in microbiota due to cage and batch effects. We focused specifically on early (one-week) and late (one-month) differences between the main factors of housing, sex, and donor type. Temporal analyses were not performed since we could not account for differences in engraftment kinetics resulting from different donor compositions. Statistical analyses were performed using analysis of variance (ANOVA) with Tukey’s *post-hoc* test for multiple comparisons using XLSTAT (version 2020.2.3; Addinsoft, Belmont, MA, USA). To resolve simple correlative relationships between microbiota composition and donor engraftment, pairwise correlations were calculated using Spearman rank correlations, to account for non-normal distribution of microbiota data, taking data pooled across all experiments. All statistics were evaluated at α = 0.05.

## Results

### Engraftment of Human Donor Microbiota

**Figure 1:**
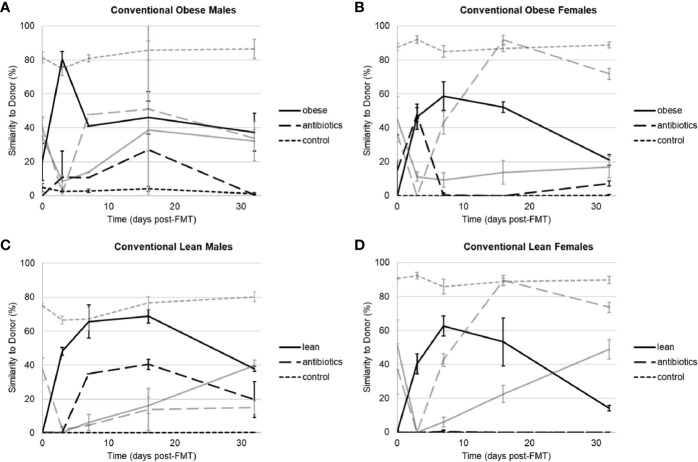
Similarity to human or indigenous mouse microbiota as determined by SourceTracker among conventionally housed mice that received microbiota from human donors, antibiotics alone, and negative control. **(A)** Male mice that received Ob1. **(B)** Female mice that received Ob1. **(C)** Male mice that received Ln1. **(D)** Female mice that received Ln1. Black lines reflect similarity to the human donor and gray lines reflect similarity to indigenous (pre-antibiotic) mouse microbial communities. Values are mean ± standard deviation (n = 2–5 mice).

Donor-specific effects of engraftment were observed among obese and lean donors, irrespective of housing or sex. When housed conventionally, mice that received microbiota from two obese donors (Ob1 and Ob3) showed high levels of engraftment within one week following gavage, reaching 80% compositional similarity to human donor microbiota, as determined using SourceTracker, in males and 59% to 95% in females (**Figure 1**, **Supplementary Table S2**). In contrast, mice that received microbiota from the second obese donor (Ob2) showed lower engraftment, reaching only 27% similarity to donor in males and 8% in females. Among mice that received microbiota from three lean donors (Ln1, Ln2, and Ln3), 1-week engraftment reached between 50% to 65% similarity to donor in males and 44% to 92% similarity in females. When engraftment was successful (>50% similarity to human donor microbiota), microbial communities in humanized groups became significantly closer to that of the respective donors following gavage, and communities in both control groups showed temporal variation (**Supplementary Figure S2**).

When data from all three obese and lean donors were compared within 1-week post-gavage, engraftment in conventionally housed mice was only significantly lower in recipients of microbiota from obese donor Ob2 (Tukey’s *post-hoc P* ≤ 0.0001). Similar findings were observed in SPF housing, except that in addition to obese donor Ob2, 1-week engraftment was also significantly lower in recipients of microbiota from lean donor Ln2 (*P* ≤ 0.0001; **Supplementary Table S2**, **Figures S3**, **S4**). Thus, among recipients of lean donor Ln2, mice housed in conventional setting had greater 1-week engraftment compared to those housed in SPF conditions (*P* ≤ 0.0001). However, differences in 1-week engraftment due to housing conditions were not observed in recipients of lean donor Ln1 or recipients of obese donors Ob1 or Ob2. Similarly, among all mice in conventional and SPF housing, differences in 1-week engraftment were not observed due to sex (*P* = 0.72).

We followed engraftment over a four-week period to evaluate stability. Similar to 1-week engraftment, mice that received microbiota from lean donor Ln2 had greater four-week engraftment in conventional housing compared to those housed in SPF conditions (Tukey’s *post-hoc P* = 0.002). Among recipients of either lean donor Ln1 or obese donor Ob1, SPF mice had greater four-week engraftment compared to conventional mice (*P* ≤ 0.0001). There were no differences in four-week engraftment between conventional and SPF mice that received microbiota from obese donor Ob2, as engraftment was poor among all recipient mice.

**Figure 2:**
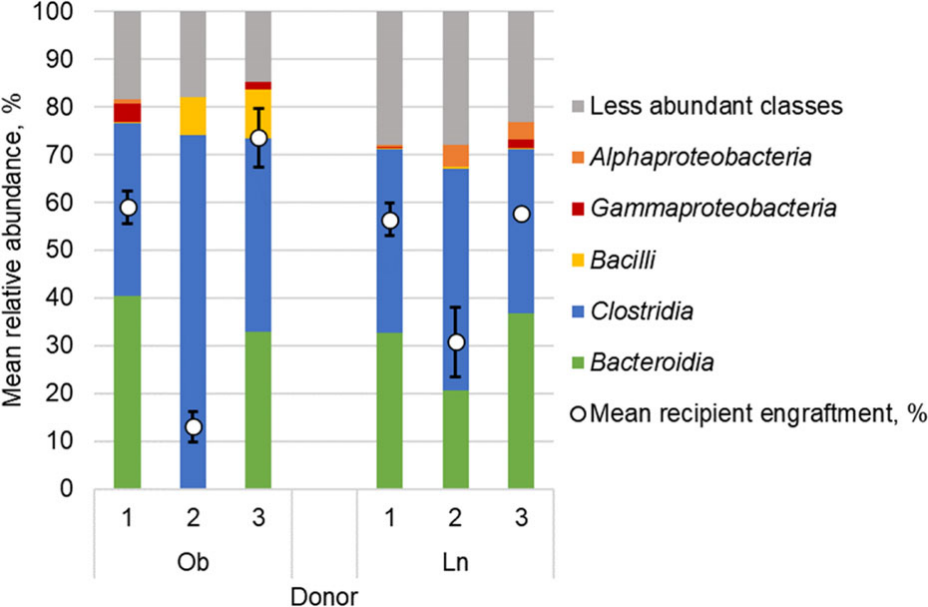
Fecal microbial composition of obese (Ob) and lean (Ln) human donors represented as mean relative abundance (%) of predominant classes. White circles reflect percent similarity to donor microbiota within 1-week among all mice that received microbiota from each respective donor as determined by SourceTracker; values are mean ± standard deviation.

We found that donor-specific effects of engraftment were associated with donor microbial community structure (**Figure 2**). When including all obese and lean microbiota recipients kept under either conventional or SPF housing, the relative abundance of microbiota from the class *Bacteroidia* in the donor sample was significantly correlated with the level of engraftment within 1-week post-gavage (Spearman’s *ρ* = 0.73, *P* ≤ 0.001; **Supplemental Figure S5**). Relative abundances of microbiota from the class *Gammaproteobacteria* were also correlated with engraftment within 1-week (*ρ* = 0.66, *P* = 0.002). In contrast, relative abundance of the class *Clostridia* was negatively correlated with 1-week engraftment (*ρ* = -0.66, *P* = 0.002).

### Obese Microbiota Is Associated With Weight Gain and Insulin Resistance

To assess the effect of microbiota transplantation on mouse phenotypes, the change in total body weight was monitored from the date of fecal microbiota transplantation (FMT) until 10-weeks post-gavage (**Table 1**). Conventionally housed mice that received microbiota from obese donor Ob1 had significantly greater weight gain relative to mice that received lean donor Ln1 or either control group, regardless of sex (Tukey’s *post-hoc P* ≤ 0.001). Similarly, male mice that received microbiota from obese donor Ob3 showed greater weight gain compared to males that received microbiota from lean donor Ln3, although this did not reach statistical significance (*P* = 0.42). However, similar weight gain was not observed in females who received the same donor Ob3 microbiota (*P* = 0.82). Moreover, mice that received microbiota from obese donor Ob2, which showed significantly poorer engraftment, did not experience a similar weight gain relative to mice that received lean microbiota in either sex (*P* = 0.93 in males, *P* = 0.66 in females).

**Table 1:** Change in total body weight (g) from baseline to 10-weeks post-gavage.

Housing/Sex	Replicate	Lean Donor		Obese Donor		*P*-value
		n	ΔWeight (g) ± SD	n	ΔWeight (g) ± SD		
**Conventional Male**	1	4	11.4 ± 5.5	3	18.1 ± 3.3	<0.01
	2	3	10.8 ± 1.9	4	11.9 ± 1.8	0.93
	3	3	12.1 ± 0.4	4	13.5 ± 1.6	0.42
**Conventional Female**	1	5	6.5 ± 1.2	5	10.0 ± 5.5	<0.01
	2	5	6.4 ± 2.1	5	7.2 ± 1.7	0.66
	3	5	7.0 ± 1.5	5	5.6 ± 1.7	0.82
**SPF Male**	1	4	11.5 ± 3.8	4	12.5 ± 2.8	0.02
	2	4	12.0 ± 2.6	4	11.5 ± 4.8	1.00
**SPF Female**	1	5	8.0 ± 1.4	5	6.9 ± 0.6	0.04
	2	5	7.5 ± 2.2	5	7.2 ± 1.5	1.00

ANOVA was performed on data from 4- to 10-weeks post-gavage between experimental and control groups. P-values reflect Tukey’s post-hoc comparison between lean and obese groups.

**Figure 3:**
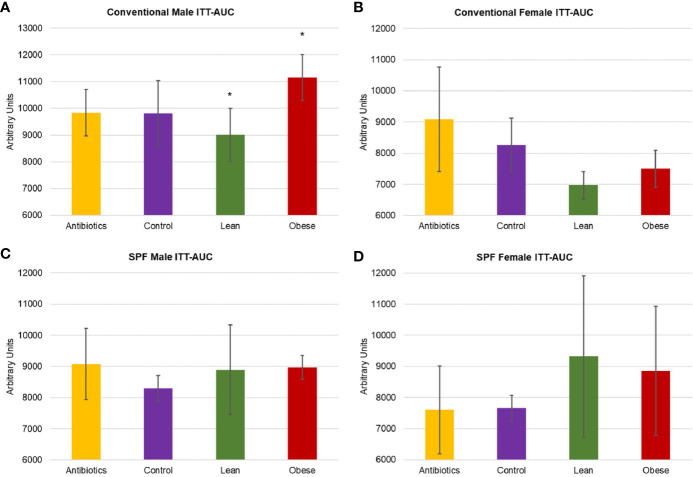
Metabolic characterization of conventionally housed and SPF mice that received microbiota from obese donor Ob1, microbiota from lean donor Ln1, antibiotics alone, and negative control. **(A, C)** Male and **(B, D)** female area under curve (AUC) of insulin tolerance test (ITT) for conventional and specific-pathogen-free (SPF) housing, respectively. Values are mean ± standard deviation (n = 2–5 mice); *P < 0.05 between recipients of microbiota from Ob1 and Ln1.

We next investigated whether FMT with microbiota from obese donors could lead to associated changes in glucose homeostasis. Insulin tolerance test (ITT) was performed at 10-weeks to evaluate insulin sensitivity (**Figure 3**). Male mice that received obese microbiota (donor Ob1) showed significantly greater insulin resistance, with 21% greater area under the curve (AUC) relative to mice that received lean microbiota (Tukey’s *post-hoc P* = 0.023). Female mice that received obese microbiota had 7% greater AUC compared to those that received lean microbiota (*P* = 0.378), but had significantly lower ITT AUC than either control group (*P* = 0.039 and 0.025, relative to antibiotics or water alone).

### Specific-Pathogen-Free Housing Blunts Phenotypic Changes

To evaluate the impact of housing conditions on the phenotypic differences, we compared SPF-housed mice that received microbiota from the same donors tested under conventional housing. The results observed in conventionally housed mice were not well reproduced in SPF mice. A significant increase in weight gain was seen in male SPF mice that received microbiota from obese donor Ob1, relative to male recipients of lean donor Ln1 (Tukey’s *post-hoc P* = 0.017). In contrast to conventionally housed mice, females who received lean donor Ln1 microbiota had unexpectedly greater weight gain than females that received obese donor Ob1 (*P* = 0.04). SPF mice that received microbiota from obese donor Ob2 did not experience increased weight gain compared to recipients of lean donor Ln2, regardless of sex (*P* = 1.00). Due to lack of significant weight gain among conventionally housed mice, the third donor pair was not replicated among SPF mice.

SPF mice that received microbiota from obese donors did not exhibit an obesity-associated insulin resistant phenotype (**Figure 3**). Although male recipients of obese donor Ob1 had greater weight gain relative to male recipients of lean donor Ln1, ITT results showed no significant differences among groups (Tukey’s *P* ≥ 0.075). Similarly, insulin resistance was not significantly different between female mice (*P* ≥ 0.415). These results suggest that mice housed in SPF conditions are less likely to exhibit phenotypic changes after microbiota transplantation.

## Discussion

The role of the intestinal microbiota in the pathogenesis of obesity and obesity-related metabolic disorders remains poorly understood due to inconsistent correlations among bacterial taxa (Finucane et al., 2014). It is imperative to establish a controlled and modifiable animal model to gain a mechanistic understanding of complex host-microbe interactions involved. In this study, we used our previously established antibiotic-based HMA model to transfer human gut microbiota into mice and compare phenotypic differences among recipients of microbiota from lean and obese human donors. We further sought to investigate the impact of housing conditions to determine if the conventional setting could represent a more clinically translatable environment than SPF condition. All housing parameters were consistent between conventional and SPF conditions, with the exception of maintenance of mouse colonies under microisolator-filtered conditions and colony manipulation done in a laminar flow hood for SPF housing.

We found that the human obese phenotype could be transmitted to conventional mice *via* microbiota transfer, however, both donor engraftment and phenotypic response were donor-dependent and, in part, reflected broad differences in microbiota composition. Engraftment was significantly greater in mice that received microbiota from donors with greater relative abundances of microbiota from the classes *Bacteroidia* and *Gammaproteobacteria*, and lower relative abundances of *Clostridia*. This is consistent with other studies that describe selective enrichment of members of the phyla *Bacteroidetes* over *Firmicutes* (Wos-Oxley et al., 2012; Hintze et al., 2014; Staley et al., 2017; Wrzosek et al., 2018). Moreover, our previous work in humans has suggested that members of the family *Bacteroidaceae* may serve as an essential scaffold to facilitate donor engraftment (Staley et al., 2019). The donor sample size used in this study, only three donors per group, was not sufficient for robust investigation of highly resolved taxonomic units (*e.g.*, amplicon sequence variants). Thus, further study is needed to validate the effects of donor composition, at more specific taxonomic resolution, on engraftment success and influence on phenotypic development. In addition, other factors including underlying metabolic diseases or demographic differences may also be related to the extent microbiota transplantation occurs in recipient mice.

Interestingly, ability to transfer obese phenotype was not reflected in stability of engraftment in recipient mice. Early engraftment was similar among mice despite housing conditions. Engraftment remained more stable in SPF mice over time compared to conventionally housed mice. However, phenotypic changes in recipient mice, when present, were seen most frequently from weeks four to ten. This finding is similar to a previous study that showed early-life administration of sub-therapeutic doses of antibiotics caused long-term changes in metabolism and adiposity (Cho et al., 2012). Thus, early changes in bacterial community organization resulting from antibiotics and/or FMT may be sufficient to drive phenotypic responses. We postulate that our repopulation with human microbiota following antibiotics offsets phenotypic effects from antibiotics, as significant increase in weight gain and insulin resistance was not observed in antibiotic-treated controls relative to untreated controls. Further, antibiotic treatment is likely to reduce cage differences in mouse microbiota resulting from different litters (Ericsson et al., 2015). Previous study in humans has suggested that repeated FMT administration may be required to achieve lasting improvement in metabolic homeostasis in the absence of antibiotic administration (Kootte et al., 2017). Further study will be necessary to understand community-level and functional responses driving phenotypic progression to improve microbiota-based interventions.

As the intestinal microbiota community as a whole is becoming recognized as a key regulator of host health (Byndloss and Bäumler, 2018), there is a critical need to reconsider how animal housing influences the resilience and impact of this community (Dobson et al., 2019). When housed conventionally, the decay of the human microbiota signature was associated with a partial recovery of the indigenous mouse microbiota, which was not observed under SPF conditions. However, weight gain was observed more consistently among obese microbiota recipients housed conventionally, with corresponding changes in glucose homeostasis, which were not observed under SPF conditions. Thus, while SPF housing allows more consistent and durable manipulation of the microbiota, challenges from exogenous microbiota may be necessary to influence phenotypically relevant shifts in microbial community organization that underly chronic conditions like obesity. Cohousing SPF mice with “dirty” feral mice resulted in a shift in the microbiota and activation of the innate and adaptive immunity to better reflect that of adult humans (Beura et al., 2016; Huggins et al., 2019), and these “dirtied” mice were subsequently more resistant to infection in a sepsis model. Therefore, studies designed to translationally characterize the functional role of the microbiota should carefully consider the environmental variable and its effect on host physiology.

Results of this study demonstrate a potentially promising HMA model for the study of obesity and metabolic disorders. Our study was limited by small numbers of mice, which limit our ability to make definitive claims regarding the effect of microbiota on phenotype. Our data suggest that donor community composition may be used as a crude predictor of human microbiota engraftment in our HMA model and that donor-dependent factors may influence the success of phenotypic recapitulation of obese phenotypes. We further note that early reorganization of the intestinal microbiota may be sufficient to drive long-term phenotype shifts. Importantly, we also demonstrate that more restrictive housing conditions likely blunt phenotypic responses associated with weight gain and insulin resistance. These results may be similarly applicable to other microbiota-associated chronic conditions and should be carefully considered when designing studies to mechanistically investigate the role of the microbiota.

## Data Availability Statement

The datasets presented in this study can be found in online repositories. The names of the repository/repositories and accession number(s) can be found below: https://www.ncbi.nlm.nih.gov/, SRP166444.

## Ethics Statement

The studies involving human participants were reviewed and approved by the Institutional Review Board, University of Minnesota. The patients/participants provided their written informed consent to participate in this study. The animal study was reviewed and approved by the Institutional Animal Care and Use Committee, University of Minnesota.

## Author Contributions

TK carried out the study, performed the data analysis, and helped draft the manuscript, HN carried out the data analysis and drafted the manuscript. ZZ assisted with the mouse work, sample collection, and sample preparation and analysis. CS designed and oversaw the study and helped draft the manuscript. All authors contributed to the article and approved the submitted version.

## Funding

HN received partial support from the Hubbard Broadcasting Foundation. This work was supported, in part, through NIH1R01DK122056-01.

## Conflict of Interest

The authors declare that the research was conducted in the absence of any commercial or financial relationships that could be construed as a potential conflict of interest.
