# Supplementary material for: Donor Microbiota Composition and Housing Affect Recapitulation of Obese Phenotypes in a Human Microbiota-Associated Murine Model
Source: Front Cell Infect Microbiol. 2021 Feb 22;11:614218. doi: 10.3389/fcimb.2021.614218 (PMC7937608; doi:10.3389/fcimb.2021.614218)
Supplement: Supplementary file 1 [file Image_1.pdf]

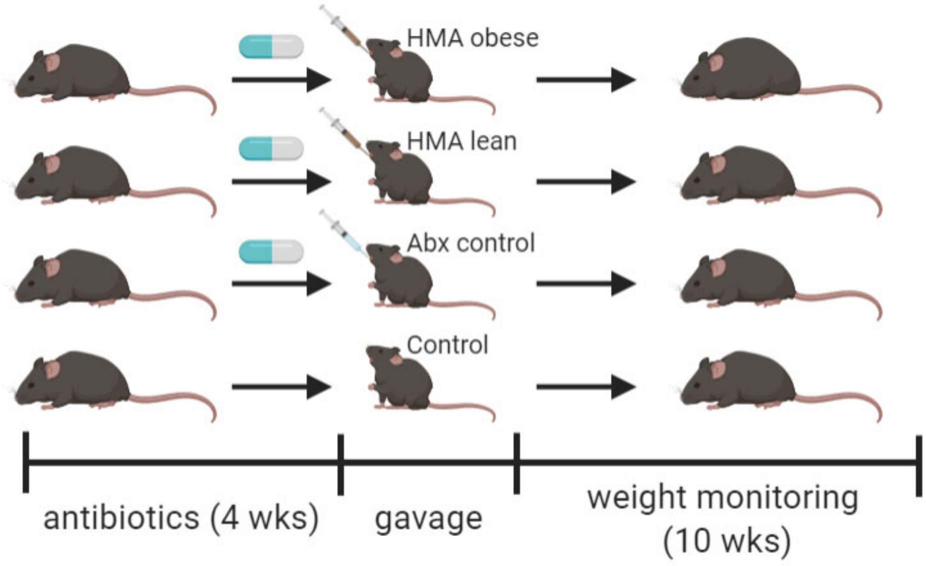

Figure S1. Study design. Mice (n = 4 males and 5 females per cohort) were randomized to undergo antibiotic conditioning and fecal microbiota transfer with either obese or lean human donor microbiota, antibiotic conditioning without fecal microbiota transfer, or neither antibiotics nor fecal microbiota transfer. Image was create using BioRender.com.
