# Supplementary material for: Donor Microbiota Composition and Housing Affect Recapitulation of Obese Phenotypes in a Human Microbiota-Associated Murine Model
Source: Front Cell Infect Microbiol. 2021 Feb 22;11:614218. doi: 10.3389/fcimb.2021.614218 (PMC7937608; doi:10.3389/fcimb.2021.614218)
Supplement: Supplementary file 3 [file Image_3.pdf]

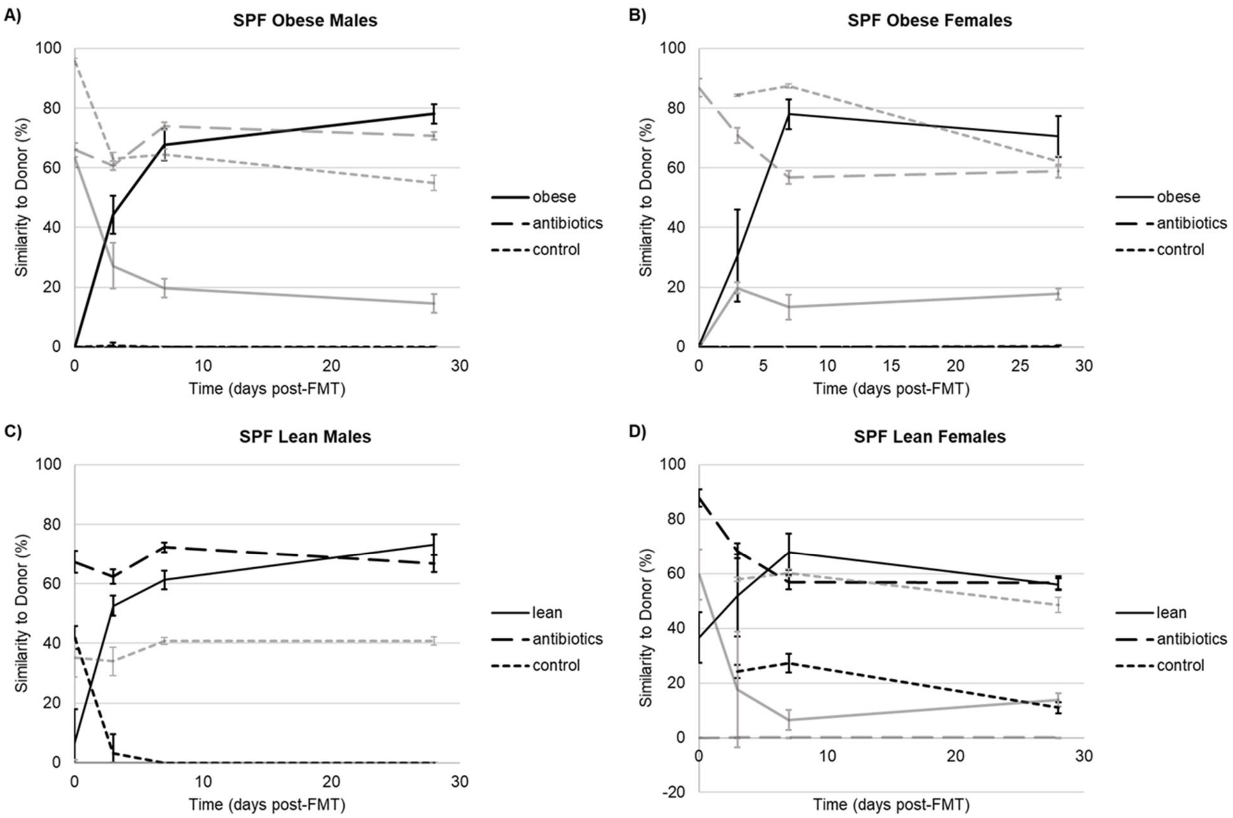

Figure S3. Similarity to human or indigenous mouse microbiota as determined by SourceTracker among mice in SPF housing that received microbiota from human donors, antibiotics alone, and negative control. A) Male and B) female mice that received Ob1. C) Male and D) female mice that received Ln1. Black lines reflect similarity to the human donor and gray lines reflect similarity to indigenous (pre-antibiotic) mouse microbial communities. Values are mean ± standard deviation (n = 2-5 mice).
