# Supplementary material for: Donor Microbiota Composition and Housing Affect Recapitulation of Obese Phenotypes in a Human Microbiota-Associated Murine Model
Source: Front Cell Infect Microbiol. 2021 Feb 22;11:614218. doi: 10.3389/fcimb.2021.614218 (PMC7937608; doi:10.3389/fcimb.2021.614218)
Supplement: Supplementary file 4 [file Image_4.pdf]

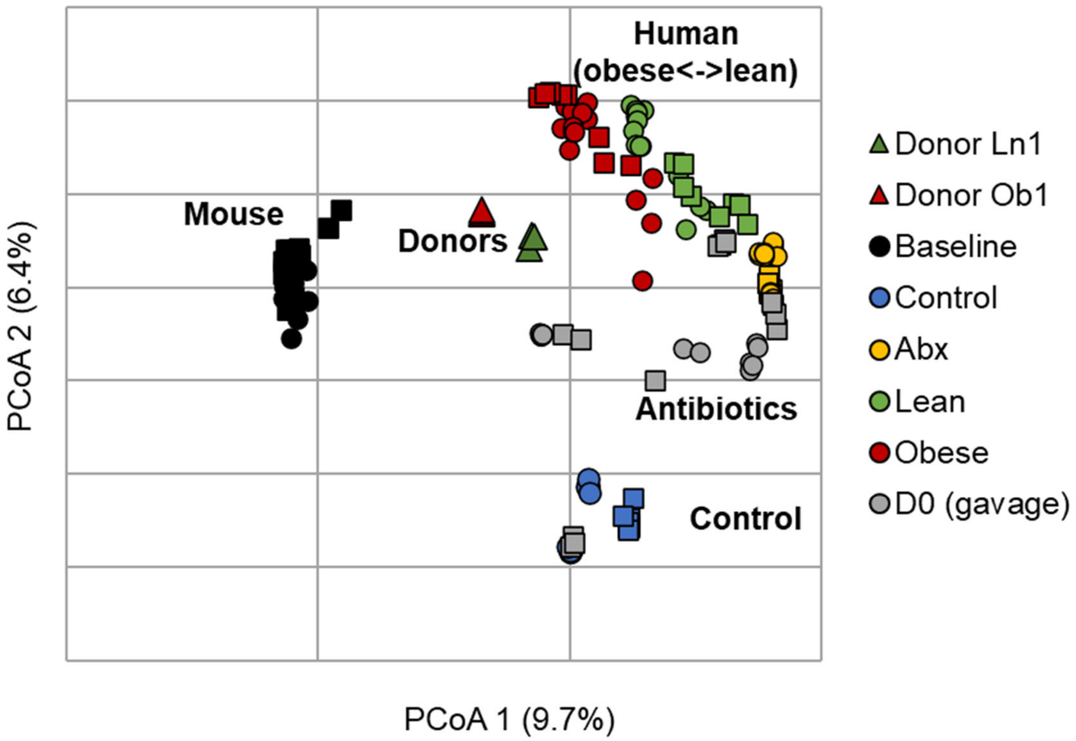

Figure S4. Principal coordinate analysis of Bray-Curtis dissimilarities among SPF mice associated with the first donor pair. Abx refers to the antibiotic control group following gavage with water. Circles (○) denote females and squares (□) denote males. Groups as denoted by color and text differed significantly in pairwise comparisons by ANOSIM (experimental R = 0.82, $P < 0.001$, Bonferroni-adjusted α=0.002).
