# Supplementary material for: Donor Microbiota Composition and Housing Affect Recapitulation of Obese Phenotypes in a Human Microbiota-Associated Murine Model
Source: Front Cell Infect Microbiol. 2021 Feb 22;11:614218. doi: 10.3389/fcimb.2021.614218 (PMC7937608; doi:10.3389/fcimb.2021.614218)
Supplement: Supplementary file 5 [file Image_5.pdf]

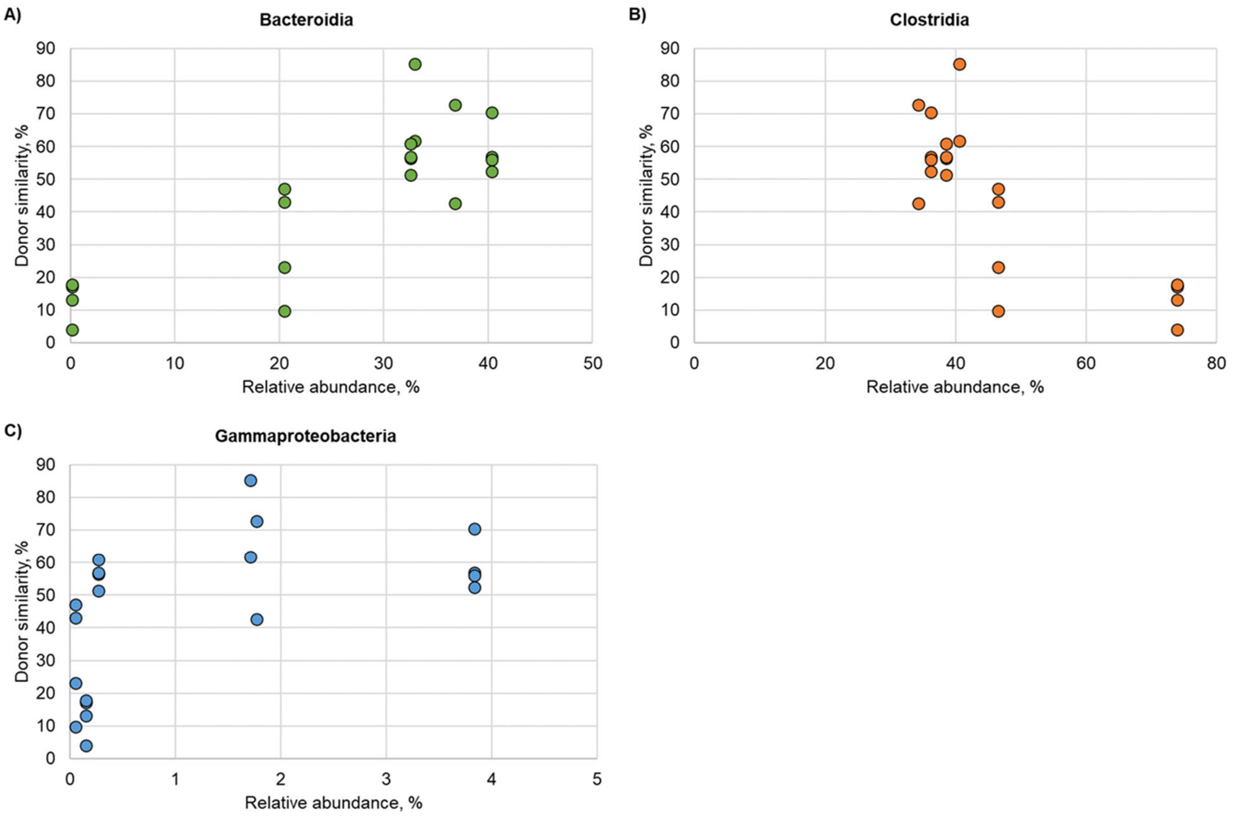

Figure S5. Correlation plots showing relative abundances of A) Bacteroidia, B) Clostridia, and C) Gammaproteobacteria and resulting engraftment in mice (determined by SourceTracker).
