# Supplementary material for: Donor Microbiota Composition and Housing Affect Recapitulation of Obese Phenotypes in a Human Microbiota-Associated Murine Model
Source: Front Cell Infect Microbiol. 2021 Feb 22;11:614218. doi: 10.3389/fcimb.2021.614218 (PMC7937608; doi:10.3389/fcimb.2021.614218)
Supplement: Supplementary file 6 [file Table_1.pdf]

Table S1. Donor demographic characteristics.

Donor	Sex	Age	BMI ($kg\ m^{-2}$)
Ln1	Male	74	22
Ln2	Male	28	22
Ln3	Male	33	21
Ob1	Female	62	32
Ob2	Male	61	49
Ob3	Female	49	46
