# Supplementary material for: Donor Microbiota Composition and Housing Affect Recapitulation of Obese Phenotypes in a Human Microbiota-Associated Murine Model
Source: Front Cell Infect Microbiol. 2021 Feb 22;11:614218. doi: 10.3389/fcimb.2021.614218 (PMC7937608; doi:10.3389/fcimb.2021.614218)
Supplement: Supplementary file 8 [file Table_3.pdf]

Table S3. Donor engraftment (mean ± standard deviation) among mice housed in SPF conditions, as determined by SourceTracker. Values sharing the same letter did not differ significantly by Tukey's *post-hoc* test ($P > 0.05$), where A had the highest engraftment and G the lowest. The final row shows all samples at all time points pooled by donor.

Sex	Time (days)	Obese		Lean	
		**Ob1**	**Ob2**	**Ln1**	**Ln2**
**Male**	**3**	$44.2 \pm 6.4^{B\text{-}E}$	$22.9 \pm 5.2^{E\text{-}G}$	$52.6 \pm 3.3^{A\text{-}D}$	10.7 ± 0.7^{FG}
	**7**	67.7 ± 5.1^{AB}	0.0 ± 0.0^{G}	61.3 ± 3.1^{AB}	$31.4 \pm 5.3^{C\text{-}F}$
	**27-32**	78.1 ± 3.2^{A}	0.3 ± 0.6^{G}	73.2 ± 3.4^{AB}	$27.4 \pm 3.0^{C\text{-}G}$
**Female**	**3**	$30.6 \pm 15.4^{C\text{-}F}$	5.3 ± 7.9^{FG}	$52.0 \pm 15.0^{A\text{-}E}$	$23.5 \pm 6.1^{D\text{-}G}$
	**7**	77.9 ± 5.1^{A}	$30.3 \pm 38.5^{C\text{-}F}$	68.0 ± 6.7^{AB}	4.2 ± 5.6^{FG}
	**27-32**	70.5 ± 6.8^{AB}	0.0 ± 0.0^{G}	56.0 ± 2.1^{ABC}	$31.8 \pm 2.6^{C\text{-}F}$
**All pooled by donor**		A	C	A	B
