# Supplementary material for: Donor Microbiota Composition and Housing Affect Recapitulation of Obese Phenotypes in a Human Microbiota-Associated Murine Model
Source: Front Cell Infect Microbiol. 2021 Feb 22;11:614218. doi: 10.3389/fcimb.2021.614218 (PMC7937608; doi:10.3389/fcimb.2021.614218)
Supplement: Supplementary file 7 [file Table_2.pdf]

Table S2. Donor engraftment (mean ± standard deviation) among conventionally housed mice, as determined by SourceTracker. Values sharing the same letter did not differ significantly by Tukey's *post-hoc* test ($P > 0.05$), where A had the highest engraftment and J the lowest. The final row shows all samples at all time points pooled by donor.

Sex	Time (days)	Obese			Lean		
		**Ob1**	**Ob2**	**Ob3**	**Ln1**	**Ln2**	**Ln3**
**Male**	**3**	80.4 ± 4.5^{ABC}	$26.5 \pm 6.0^{F\text{-}J}$	80.3 ± 36.1^{ABC}	$47.7 \pm 2.4^{C\text{-}H}$	$53.2 \pm 1.7^{C\text{-}G}$	$50.4 \pm 39.9^{C\text{-}G}$
	**7**	$40.9^{D\text{-}I}$	7.8 ± 6.8^{IJ}	$43.4 \pm 14.8^{D\text{-}H}$	$65.2 \pm 9.7^{A\text{-}E}$	$41.0 \pm 2.9^{D\text{-}I}$	$34.9 \pm 31.7^{E\text{-}J}$
	**16**	$46.1 \pm 9.7^{C\text{-}H}$	NC	NC	$68.4 \pm 4.0^{A\text{-}E}$	NC	NC
	**27-32**	$37.4 \pm 11.1^{E\text{-}I}$	0.0 ± 0.0^{J}	13.0 ± 7.0^{HIJ}	$37.3 \pm 1.5^{E\text{-}I}$	$42.1 \pm 7.7^{D\text{-}I}$	$23.6 \pm 14.4^{F\text{-}J}$
**Female**	**3**	$46.4 \pm 7.4^{C\text{-}H}$	7.9 ± 5.9^{IJ}	95.3 ± 1.2^{A}	$40.3 \pm 6.0^{E\text{-}I}$	$42.5 \pm 5.1^{D\text{-}I}$	92.3 ± 0.8^{AB}
	**7**	$58.6 \pm 8.5^{B\text{-}F}$	0.0 ± 0.0^{J}	$75.5 \pm 9.8^{A\text{-}D}$	$62.6 \pm 6.0^{A\text{-}E}$	$43.6 \pm 4.1^{D\text{-}H}$	$53.3 \pm 6.8^{C\text{-}G}$
	**16**	$52.2 \pm 3.1^{C\text{-}G}$	NC	NC	$53.2 \pm 14.1^{C\text{-}G}$	NC	NC
	**27-32**	$21.1 \pm 3.1^{G\text{-}J}$	0.0 ± 0.0^{J}	$45.4 \pm 3.3^{C\text{-}H}$	14.2 ± 1.6^{HIJ}	$35.9 \pm 3.5^{E\text{-}H}$	$39.5 \pm 2.7^{E\text{-}I}$
**All pooled by donor**		A	B	A	A	A	A

NC: sample not collected
